# Persistent and Severe Viral Replication in PBMCs with Moderate Immunosuppression Served an Alternative Novel Pathogenic Mechanism for Canine Morbillivirus

**DOI:** 10.1128/spectrum.04060-22

**Published:** 2022-12-19

**Authors:** Chuchu Feng, Yan Bu, Jiaxi Cai, Guanyu Zhao, Zishu Li, Yuening Cheng, Xiaohao Zhang, Yijun Shi, Yang Gao, Xiangnan Li, Xuexing Zheng, Xianghong Xue

**Affiliations:** a Department of Viral Infectious Diseases of Special Animals, Institute of Special Animal and Plant Sciences, Chinese Academy of Agricultural Sciences, Changchun, Jilin, China; b Jilin Provincial Key Laboratory of Special Economic Animal Molecular Biology, Institute of Special Animal and Plant Sciences, Chinese Academy of Agricultural Sciences, Changchun, Jilin, China; c College of Veterinary Medicine, Jilin University, Changchun, Jilin, China; d Department of Virology, School of Public Health, Cheeloo College of Medicine, Shandong University, Jinan, Shandong, China; e Department of Cardiology, The Second Hospital of Jilin University, Changchun, Jilin, China; f Department of Product Quality and Safety, Yantai Animal Disease Control Center, Yantai, Shandong, China; g Department of Fur Animal Business, Jilin Teyan Biotechnology Co., Ltd., Changchun, Jilin, China; Thomas Jefferson University

**Keywords:** canine distemper virus, immunosuppression, pathogenic mechanisms, viremia

## Abstract

Measles virus and canine distemper virus (CDV) cause lethal infections in their respective hosts characterized by severe immunosuppression. To furtherly acknowledge the attenuated mechanisms of the regionally ongoing epidemic CDV isolates and provide novel perspectives for designing new vaccines and therapeutic drugs, a recombinant CDV rHBF-vacH was employed with a vaccine hemagglutinin (H) gene replacement by reverse genetics based on an infectious cDNA clone for the CDV wild-type HBF-1 strain. Interestingly, unlike previously published reports that a vaccine H protein completely changed a pathogenic wild-type CDV variant to be avirulent, rHBF-vacH was only partially attenuated by alleviating the degree of viral immunosuppression, and still caused 66.7% lethality in ferrets with a prolonged period of disease. Further comparisons of pathogenic mechanisms proved that the weaker but necessary invasions into peripheral blood mononuclear cells (PBMCs) of rHBF-vacH, and subsequently persistent viral replications in PBMCs and multiple organs, together contributed to its 66.7% mortality. In addition, despite significantly higher titers than the parent viruses, rHBF-vacH would not be a suitable candidate for a live vaccine, with great invasion and infection potentials of PBMCs from 16 tested kinds of host species. Altogether, sustained and severe viral replication in PBMCs with moderate immunosuppression was first proven to be an alternative novel pathogenic mechanism for CDV, which might help us to understand possible reasons for CDV fatal infections among domestic dogs and the highly susceptible wild species during natural transmission.

**IMPORTANCE** Despite widespread vaccine campaigns for domestic dogs, CDV remained an important infectious disease in vaccinated carnivores and wild species. In recent years, the regionally ongoing epidemic CDV isolates have emphasized conservation threats to, and potentially disastrous epidemics in, endangered species worldwide. However, little is known about how to deal with the CDV variants constantly regional epidemic. In this study, we employed a recombinant CDV rHBF-vacH with a vaccine H gene replacement in a CDV wild-type HBF-1 context to attenuate the epidemic CDV variant to design a new vaccine candidate. Interestingly, rHBF-vacH was only partially attenuated by alleviating the degree of viral immunosuppression, and still caused 66.7% lethality in ferrets by weaker but necessary invasions into PBMCs, and subsequently persistent and severe viral replications in PBMCs. Significantly higher virus titers of rHBF-vacH *in vitro* might indicate the rapid cell-to-cell spreads *in vivo* that indirectly contribute to fatal infections of rHBF-vacH in ferrets.

## INTRODUCTION

Canine morbillivirus, also known as canine distemper virus (CDV), together with the measles virus (MeV), belongs to the genus *Morbillivirus* within the family *Paramyxoviridae* in the *Mononegavirales* order (1). However, unlike MeV’s strictly narrow reservoir of hosts of human and partial nonhuman primates, CDV causes a highly contagious and fatal infectious disease in a broad host range of aquatic and terrestrial animals worldwide (2, 3), with a great capacity to cross species barriers (4, 5). The CDV mortality rates vary in different susceptible species, from 0% in domestic cats, 50% in domestic dogs, to 100% in ferrets (6, 7). The wide and routine vaccination of domestic dogs has drastically reduced the incidence of canine distemper (8). However, CDV remains one of the most infectious diseases in carnivores and wild animals. Besides the immunized dogs that are occasionally infected (9 to 12), lethal CDV infections frequently have spilt in various wild animals (3, 5), such as Linnaeus’s two-toed sloths (*Choloepus didactylus*) in the United States (9), Asiatic lions (*Panthera leo persica*) in India (13), Amur tigers (*Panthera tigris altaica*) in the Russian Far East (14), the giant panda (*Ailuropoda melanoleuca*) in China (15), and the Ethiopian wolf (*Canis simensis*) in Ethiopia (16). Rapidly expanding human populations may increase domestic and stray dogs’ contact with wild animals, exacerbating the risk for disease transmission (17). Those ongoing prevalent wild-type CDV variants belonged to different genotype lineages by geographical distributions from the America-1 vaccine lineages (18). Although CDV is antigenically monotypic, defined by a polyclonal antibody over time (19), it remains an open question if the current vaccine strains confer efficiency protections against genetically divergent lineages of epidemic wild-type CDV isolates, or remain virulent in the highly susceptible species (20). Thus, it is necessary to uncover the possible pathogenic or attenuated mechanism of the epidemic CDV variants.

CDV is an enveloped negative-stranded RNA virus whose genomic RNAs are encapsidated by nucleocapsid (N) protein and then act as templates for replications and transcriptions driven by polymerase (L) protein and its cofactor phosphoprotein (P) (18, 21). The surface glycoprotein H The hemagglutinin (H) surface glycoprotein mediates viral attachment and bindings to receptors of the signaling lymphocyte activation molecule (SLAM) in lymphocytes and nectin-4 in epithelial cells (22), and determines cell tropism and viral pathogenicity (23, 24). The recently engineered CDV vaccine developments are focused on the H and another surface fusion (F) glycoprotein (22, 25, 26). The major replications of MeV and CDV occur in the SLAM-positive immune cells and then spread to the nectin-4-positive epithelial tissues (23, 27) by hematogenous transports of CDV-infected leukocytes (28), even to the central nervous system (CNS). Thus, CDV-targeted invasion into immune cells, causing severe immunosuppression in hosts, is considered the primary pathogenesis. Indeed, despite SLAM recognition, CDV V protein was also proven to sustain the swift lymphocyte-based invasion of mucosal tissue and lymphatic organs (29). Herein, to explore the pathogenic or attenuated mechanisms for the epidemic Asia-1 CDV variants, we employed a recombinant wild-type CDV rHBF-vacH with a vaccine H gene replacement. Interestingly, rHBF-vacH was only partially attenuated and led to 66.7% mortality in ferrets, with severe and persistent viral replication in PBMCs with moderate immunosuppression, posing an alternative novel pathogenic mechanism for CDV.

## RESULTS

### Constructions and identifications of rHBF-1 and rHBF-vacH.

**FIG 1:**
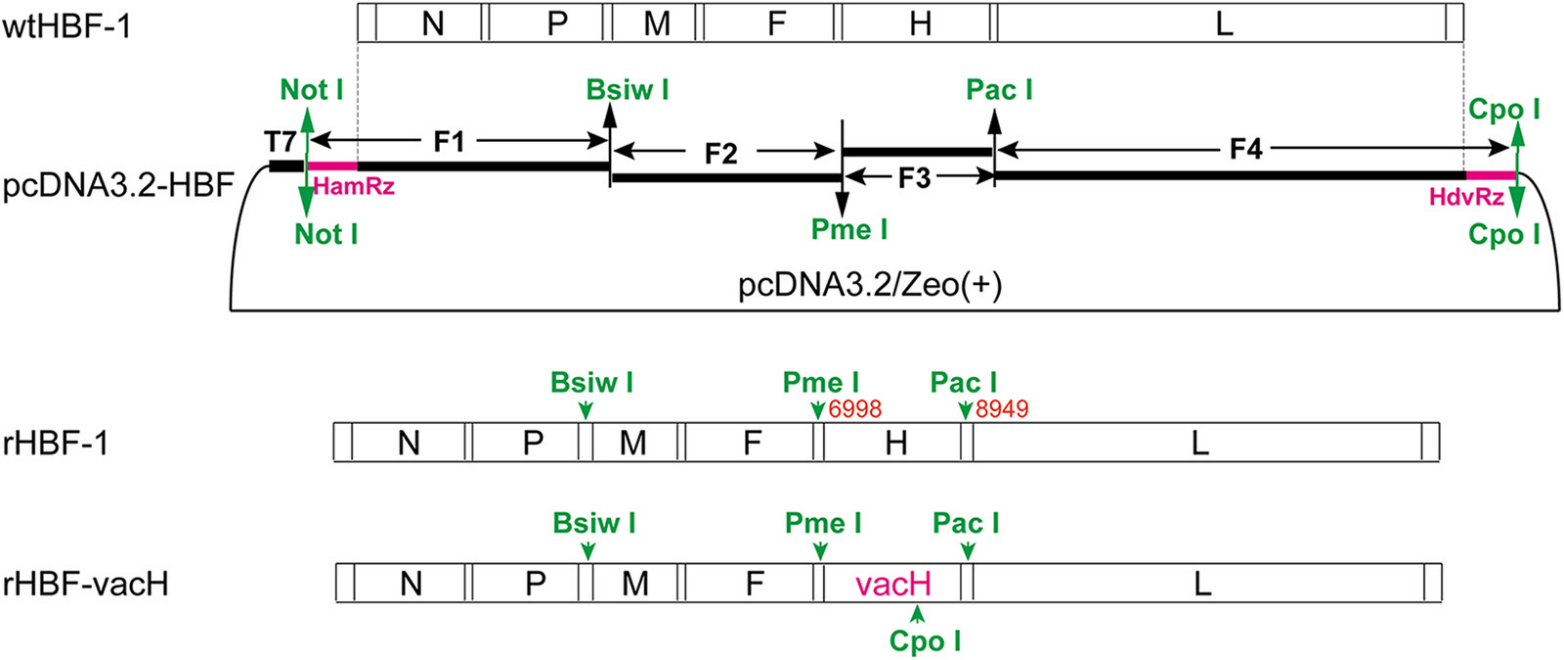
Constructions of the full-length infectious cDNA clone for the wild-type CDV HBF-1 strain and rHBF-vacH. The whole genome of the wtHBF-1 strain was artificially divided into four fragments of F1, F2, F3, and F4 based on single restriction sites of *Bsiw*I, *Pme*I, and *Pac*I. A HamRz sequence (the pink part at the beginning of the F1) and a partial HdvRz sequence (the pink part at the end of the F4) were added respectively by PCR assay. The four fragments were engineered in the modified vector of pcDNA3.2/Zeo(+) under the control of the T7 promoter by specific sites or homologous recombination, which was designated pcDNA3.2-HBF. rHBF-1 was rescued from pcDNA3.2-HBF. The H gene of wtHBF-1 was replaced by the H gene CDV vaccine strain Onderstepoort between *Pme*I and *Pac*I sites in the backbone of pcDNA3.2-HBF (data not shown), and the corresponding virus was rHBF-vacH.

The full-length antigenomic infectious cDNA clone was obtained under the control of a T7 promoter and was designated pcDNA3.2-HBF (Fig. 1), whose corresponding virus was rHBF-1. The H gene of wtHBF-1 was replaced by the H gene of the CDV vaccine strain Onderstepoort between *Pme*I and *Pac*I sites in pcDNA3.2-HBF (data not shown), and the corresponding virus was rHBF-vacH (Fig. 1).

**FIG 2:**
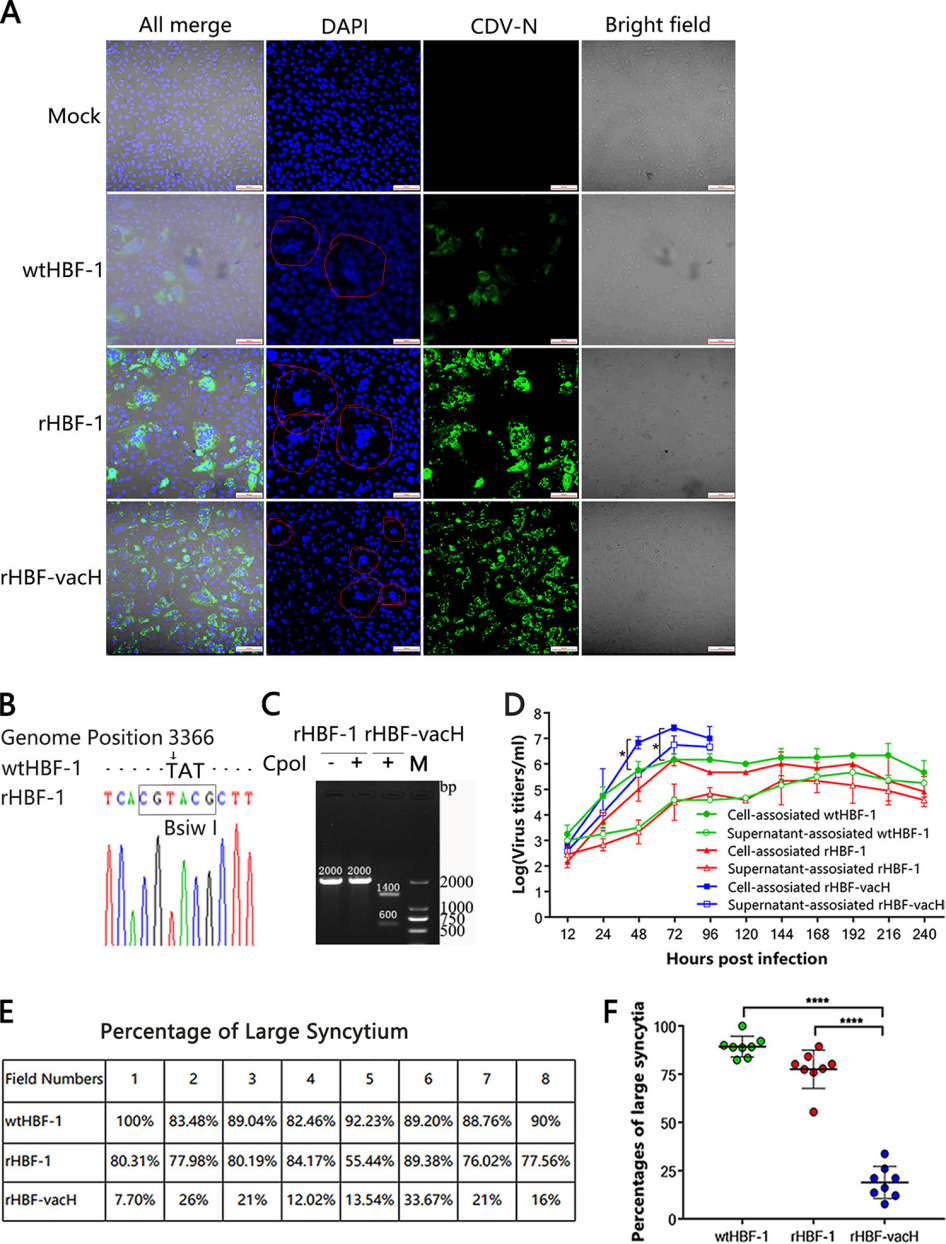
Identification and growth characteristic of the recombinant viruses. The monolayer of canine SLAM-tagged Vero cells on the slide or in a 6-well plate was infected with wtHBF-1, rHBF-1, and rHBF-vacH at 0.01 MOI for 48 h. (A) The typical syncytia and indirect immunofluorescent staining of CDVs with confocal microscopy. The cells on the slide were fixed with 4% paraformaldehyde, penetrated with 0.05% TritonX-100, and then stained with specific mouse anti-CDV-N monoclonal antibodies (1:500) and FITC-labeled goat anti-mouse IgG (1:100) as a secondary antibody, and DAPI for nuclear staining. The positive signals were observed in bright or fluorescent fields under confocal microscopy. (B) The *Bsiw*I tag identification. The TAT nucleotides in the genome of wtHBF-1 changed to GTA among 3,366 to 3,368 sites in that of rHBF-1 by sequencing. (C) *Cpo*I digestions to identify the vaccine H gene in rHBF-vacH. The *Cpo*I site was uniquely owned by the vaccine H gene. Thus, the vaccine H gene band from rHBF-vacH was digested to two small bands in Lane 3, and the band size of wild-type H gene from rHBF-1 did not change before or after *Cpo*I digestions in Lanes 1 and 2. (D) Growth characteristic of each virus. The supernatant-associated and cell-associated virus titers at the indicated time points were detected respectively by 10-serial limited dilutions. (E) The percentages of large syncytia formed by each virus in eight randomly chosen fluorescent fields. (F) The statistical analysis of the percentage of large syncytia by one-way ANOVA. All data were replicated at least three times, and the significance is labeled as *, *p* < 0.05; **, *p* < 0.01; ***, *p* < 0.001; ****, *p* < 0.0001.

The typical cytopathic syncytia of wtHBF-1, rHBF-1, and rHBF-vacH in canine SLAM-tagged Vero cells was observed in the bright fields (Fig. 2A). Similar to parent virus wtHBF-1, the recombinant viruses rHBF-1 and rHBF-vacH showed positive reactions with specific CDV-NP monoclonal antibodies (Fig. 2A) by indirect immunofluorescence assay. The *BsiwI* tag was identified that TAT nucleotides in the genome of wtHBF-1 changed as GTA among 3366 to 3368 sites in rHBF-1 (Fig. 2B). The *Cpo*I restriction site was uniquely owned by the vaccine H and was used to distinguish from the wild-type H gene. As expected, the vaccine H gene band from rHBF-vacH was digested into two small bands in Lane 3 (Fig. 2C), and the band size of the wild-type H gene from rHBF-1 did not change before or after *Cpo*I digestions in Lanes 1 and 2 (Fig. 2C).

The rHBF-1 showed similar growth characteristic with the parent virus wtHBF-1 and needed 5 or 6 days to complete stable infections. However, rHBF-vacH grew dramatically rapidly within 4 days, with 10^7.0^ 50% tissue culture infective dose (TCID_50_)/mL at 72 h postinfection (hpi) (Fig. 2D) in canine SLAM-tagged Vero cells, and completely changed the extent of syncytium formation, causing less than 25% large syncytia, whereas wtHBF-1 and rHBF-1 resulted in more than 80% large syncytia in canine SLAM-tagged Vero cells (Fig. 2E and F).

### rHBF-vacH caused (4/6) lethal infection in ferrets.

**FIG 3:**
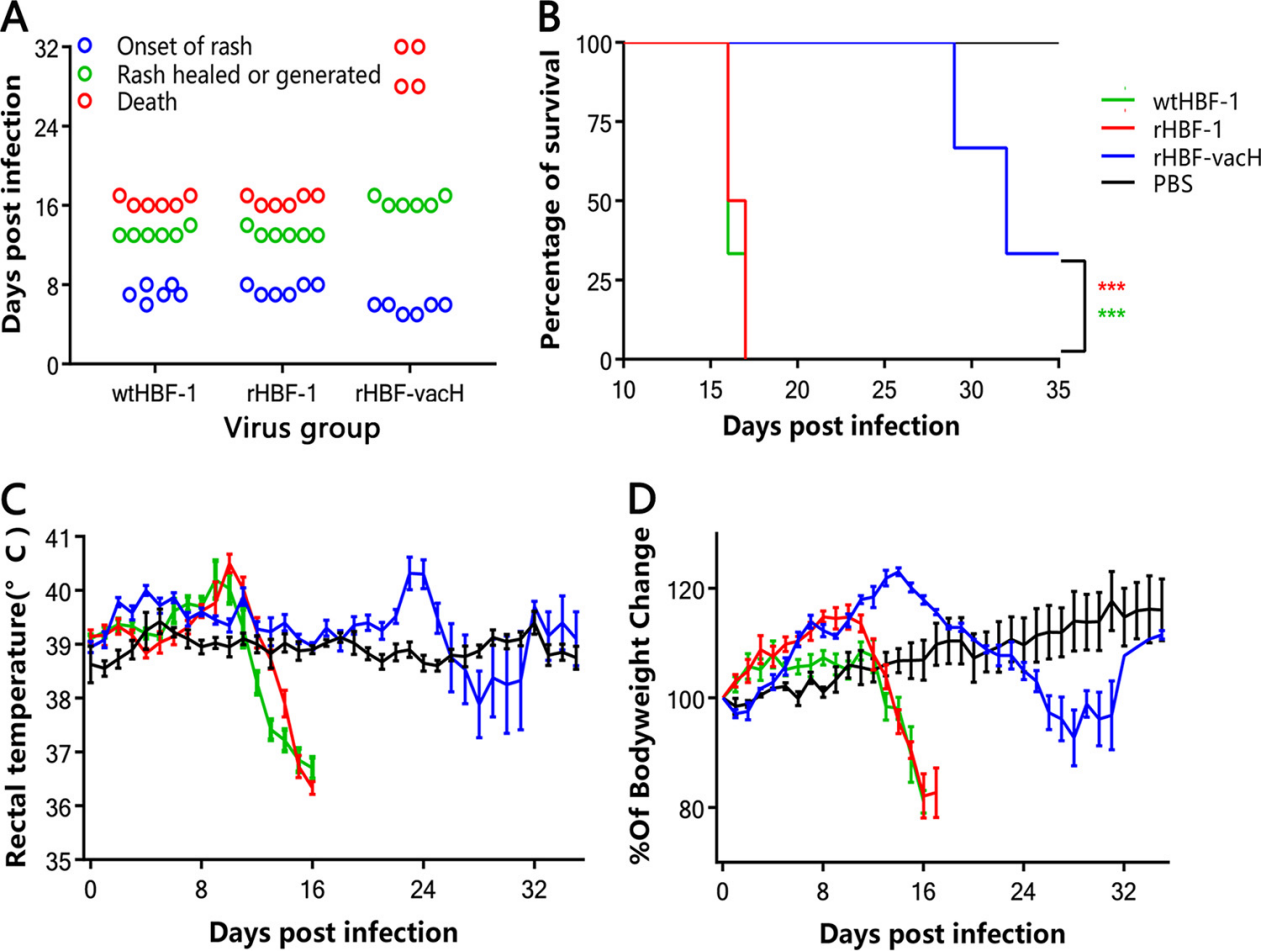
The clinical signs and pathogenesis of each virus in ferrets. Twenty-two 16-week-old male ferrets were divided into four groups (6, 6, 6, and 4 [blank group]) and infected intranasally with 10^4.5^ TCID_50_ of wtHBF-1, rHBF-1, or rHBF-vacH, respectively, under general anesthesia, and the mock-group ferrets were inoculated with PBS 0.2 mL. (A) The critical disease courses of each virus. (B) The survival rates of ferrets in each group. (C) Rectal temperatures and (D) Bodyweight changes of the four ferret groups. All data were replicated in triplicate independently and expressed as the mean ± standard deviation (SD). The significance is labeled as *, *p* < 0.05; **, *p* < 0.01; ***, *p* < 0.001; ****, *p* < 0.0001.

**FIG 4:**
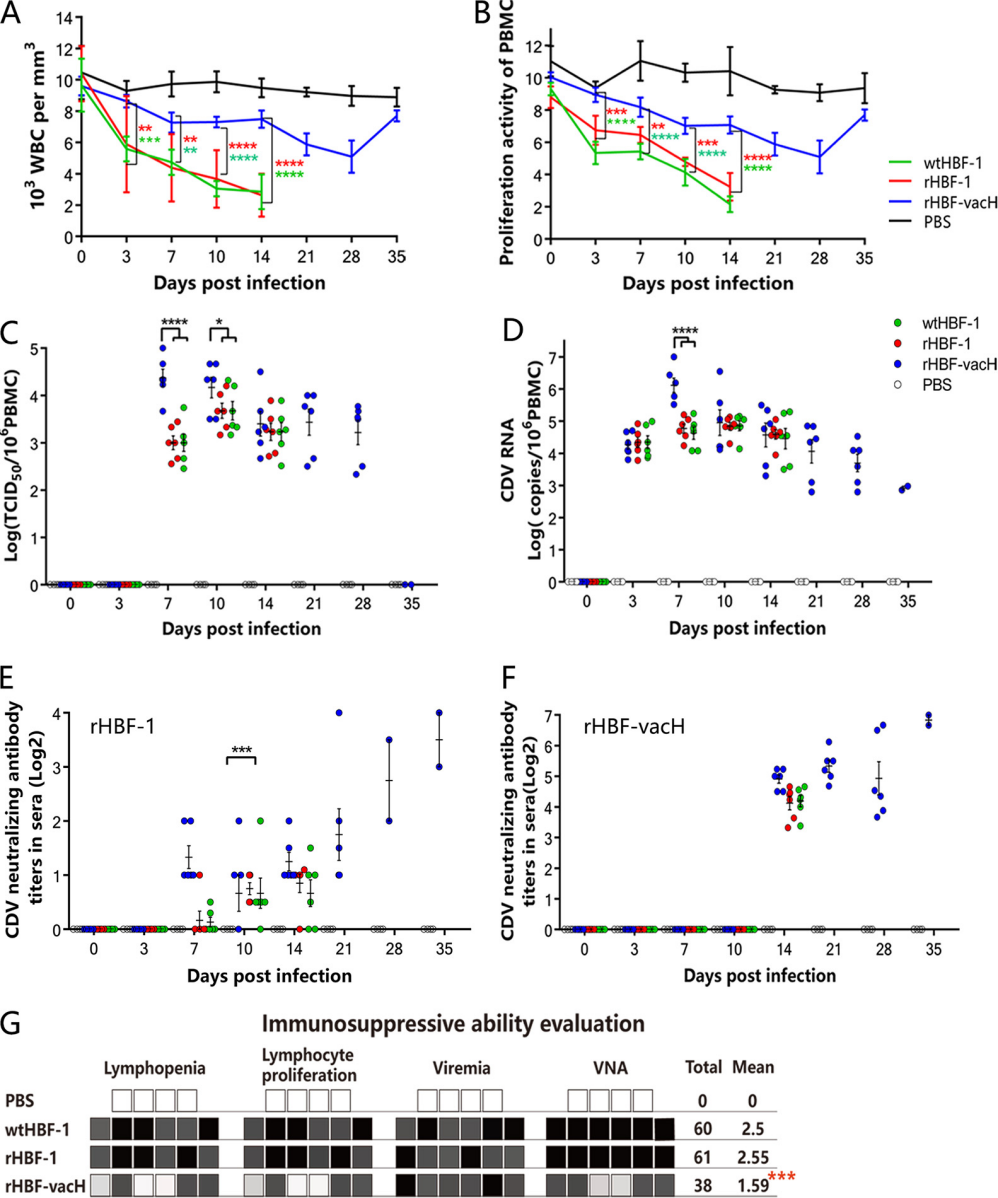
The immunosuppression evaluation of each virus in ferrets. The EDTA-treated peripheral blood samples were collected from the jugular vein of the infected ferrets at 0, 3, 7, 10, 14, 21, 28, and 35 dpi under general anesthesia and were used to evaluate immunosuppression, including the white blood cells counts, lymphocytes proliferation activity, viremia, and CDV neutralizing antibodies. (A) The white blood cell counts. Fifty microliters of EDTA-treated whole blood samples were used for white blood cell counts with an automated blood analyzer DF55 (DyMind, Shenzhen, China). (B) The lymphocyte proliferation activity. The Ficoll-purified PBMCs (2 × 10^6^) were stimulated or not stimulated with 15 μg/mL PHA, bromodeoxyuridine (BrdU) incorporation into proliferating cells was detected using cell proliferation BrdU enzyme-linked immunosorbent assay (Roche, Basel, Switzerland), and the results were read by microplate luminometer (Promega-GloMaxNavigator, WI, USA). (C) The PBMC-associated viremia. The virus titers of PBMCs (1 × 10^6^) purified by erythrocyte lysis were quantified by limiting the dilution expressed as TCID_50_/10^6^. (D) CDV RNA loadings in PBMCs (1 × 10^6^) were detected by RT-qPCR assay. (E and F) The VNAs in serum samples from the infected ferrets were determined with 100 TCID_50_ of rHBF-1 (E) or rHBF-vacH (F), respectively. (G) The immunosuppressive ability assessment of each virus in ferrets. Lymphopenia: ≥3,000 lymphocytes/μL scored 0 (white square); <3,000 lymphocytes/μL scored 1 (French gray); <2,000 lymphocytes/μL scored 2 (dark gray); <1,000 lymphocytes/μL scored 3 (black). Inhibition of lymphocyte proliferative activity: ≥80% of initial proliferation activity scored 0 (white square); <80% of initial proliferation activity scored 1 (French gray); <50% of initial proliferation activity scored 2 (dark gray); <30% of initial proliferation activity scored 3 (black). Viremia: PBMC-associated CDV titers <10^1^ TCID_50_/10^6^ PBMCs scored 0 (white square); PBMC-associated CDV titers ≥10^1^ TCID_50_/10^6^ PBMCs scored 1 (French gray); PBMC-associated CDV titers ≥10^3^ TCID_50_/10^6^ PBMCs scored 2 (dark gray); PBMCs-associated CDV titers ≥10^4^ TCID_50_/10^6^ PBMCs scored 3 (black). VNA (against the homologous virus): ≥1,000 scored 0 (white square); <1,000 scored 1 (French gray); <100 scored 2 (dark gray); <10 scored 3 (black). All data were independently replicated in triplicate and expressed as the mean ± standard deviation (SD). The statistical significance was considered at *, *p* < 0.05; **, *p* < 0.01; ***, *p* < 0.001; ****, *p* < 0.0001.

All ferrets (6/6) in wtHBF-1 and rHBF-1 groups began to have local rashes and conjunctival secretion at 7 days postinfection (dpi), aggravated about 10 dpi, developed irreversible conjunctivitis and severe generally rashes at 13 dpi, and died of CDV infections at 16 and 17 dpi (Fig. 3A and B), with the typical biphasic fevers sandwiching a transient incubation period during the initial infection stage (Fig. 3C). The rectal temperatures of ferrets infected with wtHBF-1 and rHBF-1 reached 40.1°C at 6 dpi, 40.8°C or 40.9°C at 9 to 11 dpi (Fig. 3C). In the rHBF-vacH group, the ferrets experienced a similar trend but milder fever and local rash at 6 dpi (Fig. 3A and C), and the rash disappeared around 16 dpi. However, it was interesting that the ferrets (4/6) experienced an abnormal fever, 40.2°C or 40.5°C at 22 to 24 dpi (Fig. 3C), after 10 days of rather normal rectal temperatures, the local rash vanished, and they finally died of rHBF-vacH infections at 29 dpi (2/4) and 32 dpi (2/4), respectively (Fig. 3B), with irreversible conjunctivitis and neurological signs of stiffness and twitch at intervals. The two out of six animals survived which conferred significant higher survival rate (Fig. 3B), but the 66.7% mortality of rHBF-vacH in ferrets deserves more attention. The bodyweights of the ferrets in the three virus groups were all increased at initial infection stages and began to decline after the clinical signs appeared (Fig. 3D). However, the white blood cell counts of the wtHBF-1 and rHBF-1 groups rapidly declined, and those in the rHBF-vacH group only slightly declined (Fig. 4A).

### Sustained and severe viral replication in PBMCs with moderate immunosuppression accounted for pathogenic mechanism of rHBF-vacH.

As expected, the PBMCs’ proliferation activity in wtHBF-1 and rHBF-1 groups was strongly inhibited, accompanied by leukopenia; however, there was significantly less magnitude and duration of inhibitions of PBMCs’ proliferations and leukopenia in the rHBF-vacH group (Fig. 4A and B).

Virus titers of PBMCs in the rHBF-vacH group were significantly higher at 7 dpi than those of the wtHBF-1 and rHBF-1 groups, and kept persistently high levels from 7 to 28 dpi (Fig. 4C). The viral RNA loadings in PBMCs from wtHBF-1 and rHBF-1 groups reached 10^4^ copies at 3 dpi and peaked at 7 dpi. However, the viral loadings in PBMCs of the rHBF-vacH group were significantly higher at 7 dpi and remained rather high during the whole disease course (Fig. 4D). These findings were consistent with the PBMC-associated viremia. The virus neutralizing antibodies (VNAs) in serum samples from the infected ferrets could react with homologous and heterologous viruses (Fig. 4E and F). wtHBF-1 and rHBF-1 elicited little detectable neutralizing antibody (less than 1:10) against rHBF-1 (Fig. 4E), whereas they had higher levels of neutralizing antibody against the heterologous virus of rHBF-vacH (Fig. 4F), indicating that (i) wtHBF-1 and rHBF-1 caused lethal infection inducing little humoral responses and (ii) the vaccine strain and wild-type CDV shared considerable neutralizing epitopes in their H protein, and F protein can also induce neutralizing antibody production. The levels of neutralizing antibody elicited by rHBF-vacH were higher against the homologous virus of rHBF-vacH (less than 1:100) than the heterologous virus of rHBF-1, indicating that a vaccine H protein conferred protective humoral immune responses.

Altogether, the immunosuppressive ability of each virus was evaluated with the above-mentioned four parameters. wtHBF-1 and rHBF-1 demonstrated a significantly stronger immunosuppressive ability than rHBF-vacH (Fig. 4G). Thus, rHBF-vacH caused lethal infections by continuous viral replications in PBMCs with moderate immunosuppression.

### Viral shedding and viral replications in multiple organs.

**FIG 5:**
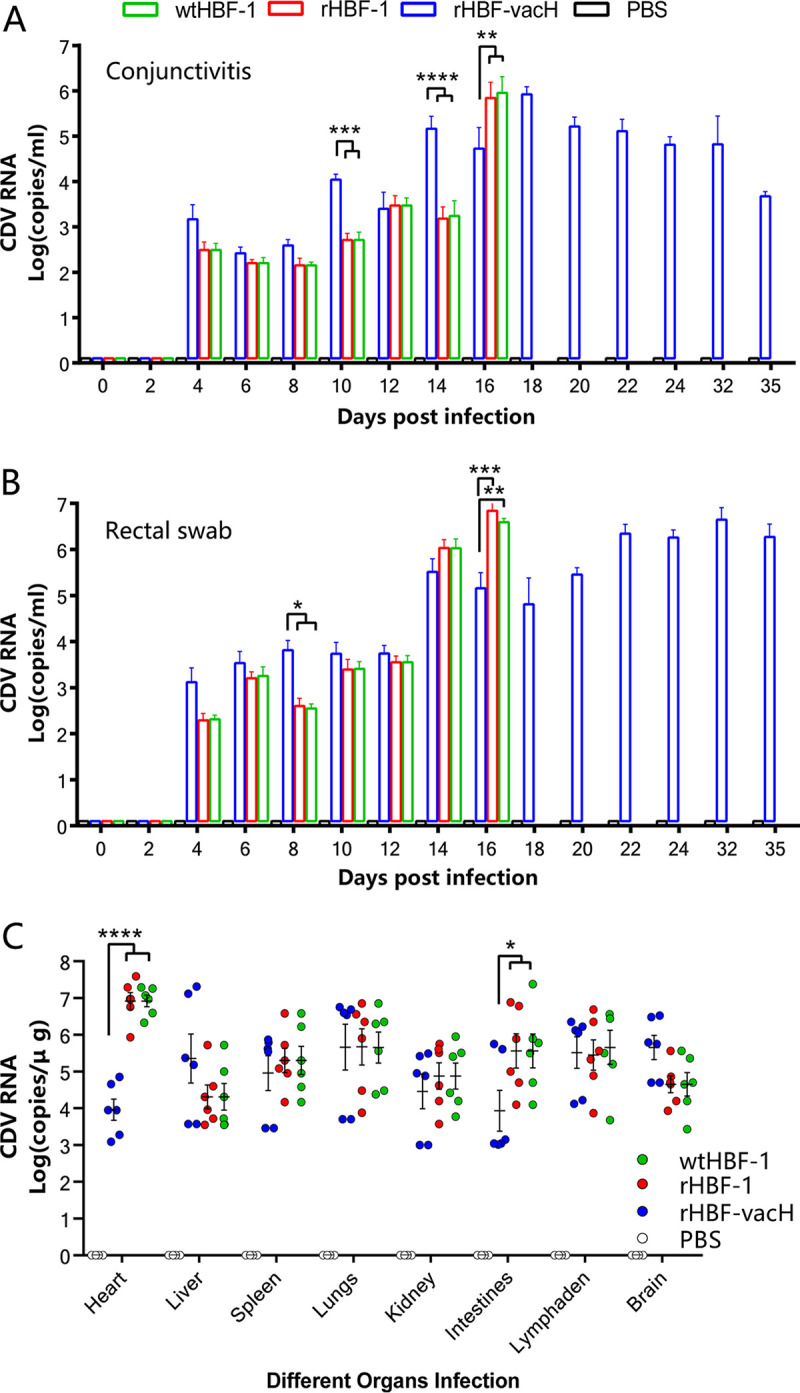
Viral shedding from conjunctiva and recta, and viral replications in multiple organs. (A) Viral shedding in conjunctiva. (B) Viral shedding in recta. The conjunctiva and rectal swabs were collected every 2 days up to 35 dpi and processed to detect viral shedding by RT-qPCR assay with CDV N gene-specific primers and probe. (C) Viral replications in multiple organs. The ill ferrets were euthanized, the nondiseased animals were euthanized at 35 dpi, and the hearts, lungs, livers, spleens, kidneys, intestines, mesenteric lymph nodes, and brains were harvested to detect viral replication. Each set of data was independently replicated in triplicate and expressed as the mean ± standard deviation (SD). The statistical significance was considered at *, *p* < 0.05; **, *p* < 0.01; ***, *p* < 0.001; ****, *p* < 0.0001.

The viral N gene copies in conjunctival and rectal swabs were positive from 4 dpi (Fig. 5A and B) before onset of clinical signs, indicating that CDV spread rapidly from infected PBMCs to general organs through blood circulation and was excreted from epithelial cells. The rHBF-vacH-infected ferrets released significantly larger amounts of viruses than ferrets in the wtHBF-1 and rHBF-1 groups at initial infection stages (Fig. 5A and B) and demonstrated continued virus releasing in the whole disease course (Fig. 5A and B). The most viral amounts were released by ferrets in the wtHBF-1 and rHBF-1 groups when they approached death (Fig. 5A and B). Viral loading in different organs of ferrets demonstrated that the three tested CDV could distribute and replicate in various organs (Fig. 5C). wtHBF-1 and rHBF-1 were easier to replicate in the heart and intestines, whereas rHBF-vacH was slightly more proliferation in the brain (Fig. 5C), which was indirect evidence of clinical neurological signs observed in ferrets that died of the rHBF-vacH infection.

### Efficient invasion of rHBF-vacH into PBMCs *in vitro* contributed to its pathogenicity in ferrets.

**FIG 6:**
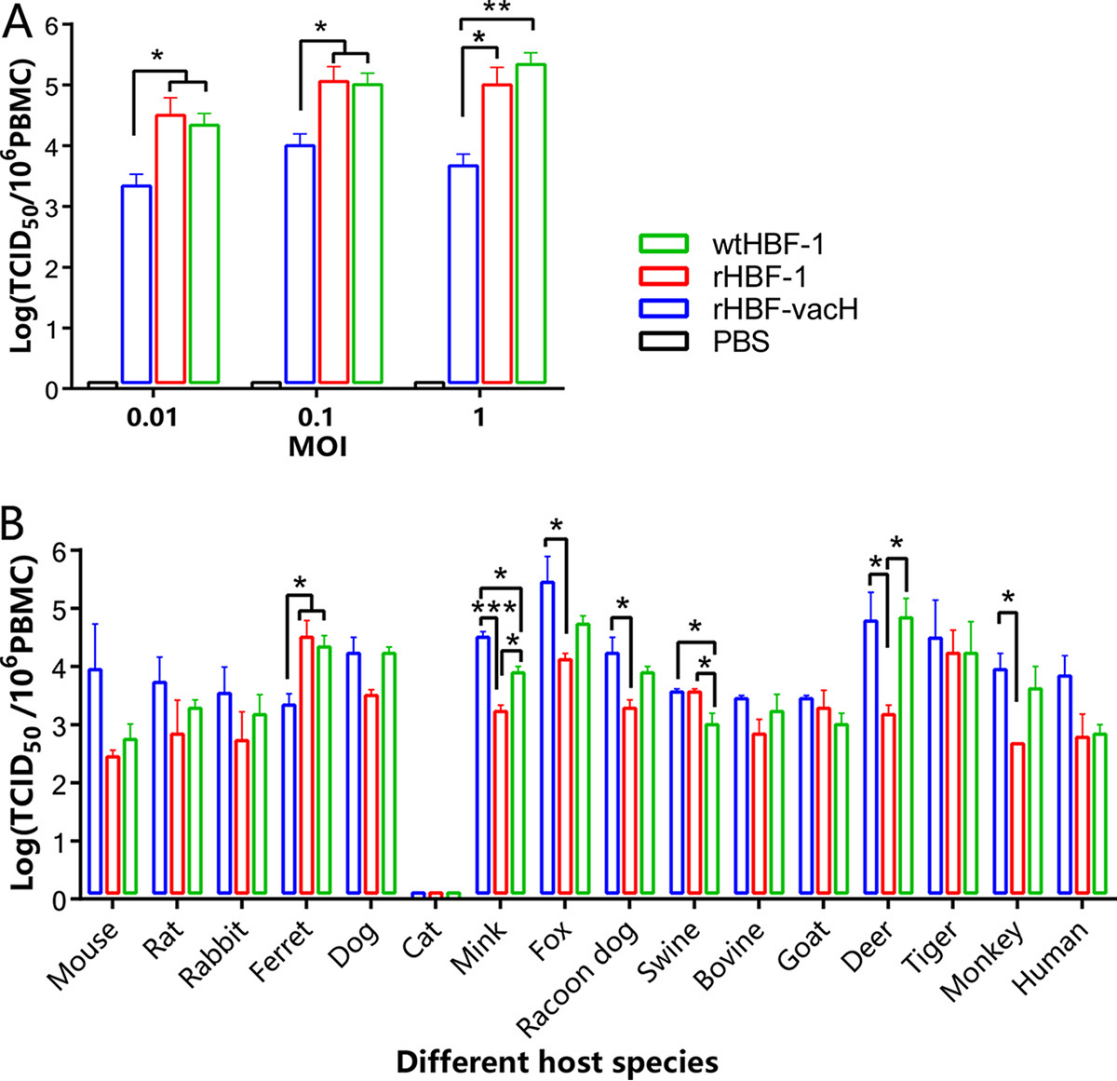
The invasion abilities of each virus into PBMCs of ferret and multihost species. The extracted PBMCs (1 × 10^6^) from the noninfected ferrets and other multihost species were experimentally infected with the three viruses for 2 h at the indicated MOI *in vitro* and were used to determine virus titers in canine SLAM-tagged Vero cells after PBS washing 3 times. (A) The correlation of invasion abilities of each virus into PBMCs of ferrets, with virus amount. (B) The invasion abilities of each virus into PBMCs of different host populations. They included insusceptible experimental animal models (BALB/c mice, rats, and Japanese white rabbits), the susceptible species (dogs, foxes, racoon dogs, minks, cats, tigers, and nonhuman primates [Rhesus macaques]), the livestock animals (pigs, goats, and cattle) and ruminants (*Cervus nippon*), and human beings. All data were independently replicated in triplicate and expressed as the mean ± standard deviation (SD). The statistical significance was considered at *, *p* < 0.05; **, *p* < 0.01; ***, *p* < 0.001; ****, *p* < 0.0001.

To get insights into the pathogenic mechanisms of rHBF-vacH, the PBMCs were experimentally infected with each virus *in vitro*. The three viruses could invade the PBMCs at divergent multiplicities of infection (MOIs) of 0.01, 0.1, or 1, and the wtHBF-1 or rHBF-1 showed significantly higher virus titers than rHBF-vacH (Fig. 6A), indicating more invasions. In the results above, we had proved that rHBF-vacH got similar viral replication in PBMCs to wtHBF-1 and rHBF-1 *in vivo* (Fig. 4A, C, and D) but showed significantly weaker immunosuppressive ability (Fig. 4G). Therefore, it was shown that less but necessary viral invasions into PBMCs were preceded by persistent and severe viral replications in PBMCs during rHBF-vacH infection (Fig. 6A).

### rHBF-vacH gained a stronger invasion capability into PBMCs of multiple species.

Furthermore, to screen the different invasion capabilities and potential infection risks of rHBF-vacH in various host species, we employed the PBMCs isolated from 16 kinds of hosts to infect with the three indicated viruses. They could efficiently invade the PBMCs of the tested multihost species (Fig. 6B), validating that CDV could infect a very broad range of hosts and easily cross species barriers (18). Our findings also proved that it could efficiently invade PBMCs of humans, consolidating evidence of potential zoonoses (5). However, unlike the weakest invasion of rHBF-vacH in ferrets, rHBF-vacH demonstrated significantly stronger capability to invade PBMCs in many tested species of mouse, mink, fox, deer, and rhesus monkey, as wells as humans, than wtHBF-1 and rHBF-1 (Fig. 6B). Our unpublished studies proved that wtHBF-1 led to fatal infection in beagles, foxes, and raccoon dogs, which indicated that more attention should be paid to the potential infection and transmission risks of rHBF-vacH when designing new live vaccine strategies. The three viruses did not invade the PBMCs in domestic cats (Fig. 6B); there were no reports of CDV morbidity and mortality in cats. However, various reports proved that CDV caused lethal infections in the big cats, tigers (Fig. 6B) and lions (24).

## DISCUSSION

### The epidemic fatal CDV variants were geographically clustered to lineages different from the vaccine strains.

Recently, many CDV infection cases in vaccinated dogs and spillover in wild animals were reported (9, 12, 16). Phylogenetic analysis revealed that the epidemic CDV isolates clustered to geographically distributed lineages other than the vaccine lineage of America-1 or America-2, which raised the question as to protection efficiency of vaccine strains (11). The high diversity of H protein conferred important roles in site mutations and antigenic drifts (30). The amino acid sequence alignment results based on the H protein of wtHBF-1 and the Onderstepoort strain demonstrated that 57 residues were different (data not shown).

### The vaccine H protein in rHBF-vacH completely changed syncytia sizes with higher virus titers.

The wtHBF-1 and rHBF-1 strains formed large syncytia in canine SLAM-tagged Vero cells with considerably similar growth characteristics, which is consistent with previous studies that a recombinant virus derived from cDNA retained the similar growth and pathogenic characteristics of its parental virus (31). However, the rHBF-vacH strain replicated rapidly and peaked 10^7.0^ TCID_50_/mL at 72 hpi in canine SLAM-tagged Vero cells, which was significantly higher than the parental wtHBF-1 and rHBF-1 strains. Importantly, rHBF-vacH completely demonstrated the small syncytia in canine SLAM-tagged Vero cells. In addition to receptor binding, H protein also provides fusion support, which is associated with conformational changes that in turn trigger fusion mediated by the F protein (23, 32). The H and F protein, together with SLAM receptor, mediated virus fusions to the cell membrane, but the H protein predominantly determined the fusogenicity, growth characteristics, and tropism of the recombinant viruses (33). Compared with wtHBF-1 and rHBF-1, rHBF-vacH gained significantly less invasions of PBMCs *in vitro* but significantly more persistent replications in PBMCs in ferrets across the disease course. The vaccine H protein might confer rHBF-vacH rapidly cell to cell spread based on the facts of rHBF-vacH rapid propagation and higher titers in canine SLAM-tagged Vero cells. For CDV, the proficiency of syncytium formation varies among different strains and correlates with the degree of viral attenuation: the more attenuated a strain is, the higher its fusogenicity (33). wtHBF-1 and rHBF-1 caused 100% (6/6) ferret death with large syncytia in canine SLAM-tagged Vero cells, and rHBF-vacH killed 66.7% (4/6) of ferrets with small syncytia in the same cells, which revealed that the pathogenicity and sizes of syncytia were not positively a negative correlation.

### rHBF-vacH was partially attenuated with a vaccine H protein replacement by relieving immunosuppression, and still led to 66.7% lethality in ferrets by severe and sustained viremia.

A wild-type virus 5804P reproducing the vaccine strain N-glycosylation pattern remained lethal in ferrets but with a prolonged course of disease, while introduction of the vaccine H protein in the wild-type context of the 5804P strain resulted in complete attenuation in ferrets (34). Our results found that a vaccine H protein in rHBF-vacH caused moderate lymphopenia and inhibition of lymphocyte proliferation, which was consistent with the above findings, but rHBF-vacH led to fatal infections in 4 of 6 ferrets, which contradicts pervious findings.

The consequences of infectious disease in animals depend on the battles between virulent pathogens and the host immune responses. In CDV-infected dogs and ferrets, the extent of lymphopenia, lymphocyte proliferation activity, viremia, and VNAs were critical indicators for degrees of immunosuppression and animal outcomes. Survivors produce robust innate and adaptive immune activation and experience transient immunosuppression and inhibition of lymphocyte proliferation, and controlled cell-associated viremia, while the CDV-killed animals experience severe leukopenia and complete loss of PBMC proliferation activity and are unable to activate immune responses to control the virus (35, 36). The wtHBF-1- and rHBF-1-infected ferrets developed typically severe lymphopenia along with dramatic loss of PBMC proliferation activity. However, similar but significantly milder lymphopenia and proliferation activity were observed in rHBF-vacH-infected ferrets. Meanwhile, PBMC-associated virus titers in wtHBF-1 and rHBF-1 groups maintained 10^3.5^ TCID_50_/10^6^ PBMCs, and steadily over 10^3.5^ TCID_50_/10^6^ PBMCs in rHBF-vacH groups until 28 dpi, which were consistent with CDV RNA loadings of PBMCs in the three groups. However, rHBF-vacH elicited high levels of VNAs in all infected ferrets, and much higher levels of VNAs in the two surviving ferrets, but wtHBF-1 and rHBF-1 elicited little detectable VNAs. Thus, the rHBF-vacH lethal infections were mainly caused by significantly severe and sustained replications in PBMCs, but not by severe immunosuppression. Taken together, novel CDV pathogenesis should suggest the following: (i) It is severe viremia, not severe immunosuppression, that is indispensable for CDV fatal infection. (ii) The severe viremia could be independent of severe immunosuppression during CDV fatal infection. (iii) It should be considered whether severe and sustained viremia might account for ongoing CDV fatal infections among domestic dogs and the highest susceptible wildlife species during natural transmissions.

### wtHBF-1, rHBF-1, and rHBF-vacH demonstrated efficient invasions into PBMCs of multihost species posing great potential infection risk.

The interaction of pathogens and host cells was mediated by viral attachment H proteins and the SLAM receptors expressed on PBMCs. The capability of invasion into PBMCs of ferrets mediated by the avirulent H protein in rHBF-vacH was not as powerful as the virulent H protein in wtHBF-1 and rHBF-1, but it provided rHBF-vacH efficient invasions into PBMCs, a critical prerequisite for its later sustained lethal replications. The rHBF-vacH gained necessary invasions of PBMCs, persistent replications and generally spread, resulting in lethal infections in ferrets, which raised a novel controversy concerning total attenuation of wild-type CDV isolates with a vaccine H protein replacement published previously.

Oligosaccharides on the CDV H protein may influence the strength of the interactions with cellular receptors, and alternatively influence the extent of viral propagation by altering the fusion efficiency of the F-H protein complex expressed in infected cells (33, 34). The H protein of the wtHBF-1 and rHBF-1 strains had more potential glycosylation sites than the vaccine H proteins in rHBF-vacH (data not shown), which might explain its weaker invasion into PBMCs *in vitro*. However, the invasions of wtHBF-1, rHBF-1, and rHBF-vacH into PBMCs of multihost species displayed inconformity. rHBF-vacH showed universally stronger invasion into PBMCs in the tested hosts than wtHBF-1 and rHBF-1, which indicated that rHBF-vacH might lead to fatal infection in a broad range of hosts like the parental wtHBF-1 and rHBF-1.

In conclusion, our findings demonstrated that the wild-type CDV HBF-1 strain in Asia-1 was partially attenuated with a vaccine H protein replacement by relieving the degree of immunosuppression, and other viral proteins indispensably determined its pathogenicity. The severe sustained viral replication in PBMCs with moderate immunosuppression accounted for lethal infection of rHBF-vacH, which proposed an alternative novel pathogenic mechanism for CDV in natural cross-infection cases among domestic dogs and the most susceptible wildlife species. In addition, rHBF-vacH was not suitable for a new live vaccine since it universally invaded PBMCs of multihost species, potentially posing great fatal infection risks.

## MATERIALS AND METHODS

### Cells, viruses, ferrets, and blood samples.

The Vero cells (CCL-81), the canine SLAM-tagged Vero cells (37), and the BSR-T7/5 cells (38) were maintained in Dulbecco’s modified Eagle’s medium (DMEM) supplemented with 10% heat-inactivated fetal bovine serum (FBS) (TransGen, Beijing, China), penicillin (100 U/mL) (Gibco, CA, USA), and streptomycin (100 μg/mL) (Gibco, CA, USA) at 37°C in a 5% CO_2_ incubator. The CDV HBF-1 strain (wtHBF-1) was adapted in SLAM-tagged Vero cells from the wild-type CDV Hebei (KC427278) isolated strain. CDV vaccine strain Onderstepoort-os was propagated in Vero cells. The CDV-NP monoclonal antibody was purchased from Veterinary Medical Research & Development (VMRD, WA, USA). Twenty-two unvaccinated male ferrets (Mustela putorius furo) aged 16 weeks were purchased from Wuxi Coral Reef Biotechnology Co., Ltd. (Wuxi, China).

The EDTA-treated blood samples (*n* ≥ 3 in each animal) were collected from the experimental BALB/c mice, rats, and Japanese white rabbits; from healthy dogs (beagles, *Canidae*), foxes (Blue fox, *Canidae*), racoon dogs (Wusuli raccoon dog, *Canidae*), minks (standard black mink, *Mustelidae*), and deer (Cervus Nippon, *Cervidae*) in fur animal farms and a deer farm in Jilin Province; from healthy cats (Chinese Garden cat, *Felidae*) in a pet hospital in Changchun City; from livestock animals healthy pigs (landrace, *Suidae*), goats (Boer goat, *Bovid*), and cattle (Chinese Simmental cattle, *Bovid*) in their respective farms in Jilin Province; from tigers (Manchurian tiger, *Felidae*) in the Siberian Tiger Zoo in Changchun City; from healthy nonhuman primates (Rhesus macaques, *Cercopithecidae*) in a monkey breeding farm in Beijing City; and from four healthy human volunteers (*Hominidae*) of 30 to 40 years old at the Institute of Special Animal and Plant Sciences in Changchun City.

All the animal studies and sample preparations were approved by the Institutional Animal Care and Use Committee of the Institute of Special Animal and Plant Sciences (ISAPS-2020-062) and complied with the Animal Ethics Procedures and Guidelines.

### Full-length genomic infectious cDNA clone.

The artificial multiple cloning sites of *Not*l, *Bsiw*I, *Pme*I, *Pac*l, and *Cpo*I and partial sequences of hepatitis delta virus ribozyme (HdvRz) were synthesized and inserted at the *Pme*I site in pcDNA3.1/Zeo(+) (Invitrogen, CA, USA) and were designated pcDNA3.2/Zeo(+). Total RNAs in wtHBF-1-infected canine SLAM-tagged Vero cells were prepared with the RNeasy minikit (Qiagen, Dusseldorf, Germany) to synthesize the first-strand cDNA with the Superscript III First-Strand Synthesis SuperMix (Invitrogen, CA, USA). Four paired specific primers (Table 1) covering the full-length genome of wtHBF-1 were used to amplify four long fragments of F1, F2, F3, and F4. The purified four long fragments were first cloned to pEASY-Blunt or Simple vectors, and then spliced gradually into pcDNA3.2/Zeo(+) with T4 DNA ligase or homologous recombination. A hammerhead ribozyme sequence (HamRz) (bold in Table 1) was added upstream of F1 by PCR as partial forward primers, and partial sequences of HdvRz (underlined in Table 1) were added downstream of F4 by PCR as partial reverse primers. Finally, a full-length antigenomic cDNA clone for the wtHBF-1 strain was generated under the control of a T7 promoter and was designated pcDNA3.2-HBF (Fig. 1). The H gene of the CDV vaccine Onderstepoort strain was amplified with vacH-for/rev primers (Table 1), and was used to replace the H gene of the wtHBF-1 strain between *Pme*I and *Pac*I sites in pcDNA3.2-HBF, and the resultant plasmid was designated pcDNA3.2-HBF-vacH (data not shown). Meanwhile, the open reading frames of N, P, and L genes of the wtHBF-1 strain were amplified, respectively, with specific primers (Table 1) and cloned into pcDNA3.1/Zeo(+), yielding three helper plasmids of pcDNA3.1-N_HBF_, pcDNA3.1-P_HBF_, and pcDNA3.1-L_HBF_.

**TABLE 1:** Primers used for constructions of the indicated plasmids^*a*^

Primer name	Primer sequences (5′–3′)	Nucleotides positions in the genome of wtHBF-1
F1-for	*GCGGCCGC*TGTTAAGCGTCTGATGAGTCCGTGAGGACGAAACTATAGGAAAGGAATTCCTATAGTCACCAGACAAAGTTGGCTAAG	1–20
F1-rev	G*CGTACG*TGAAAGCAGTTTTGAGCCT	3351–3377
F2-for	CTGCTTTCA*CGTACG*CTTAAAAGCAATTATAAAAAACTTAGG	3360–3420
F2-rev	*GTTTAAAC*GCTTTTGAAGGAAATTAGGCGGGACT	6972–7006
F3-for	TTCCTTCAAAAGC*GTTTAAAC*TGCAACAAATAGTGGCG	6985–7022
F3-rev	GGA*TTAATTAA*CACGGTCATCATCCCTCAGTTCAATTGA	8922–9861
F4-for	GGGATGATGACCGTG*TTAATTAA*TCCCTTACCGATGATTGAATT	8935–8978
F4-rev	CGGATGCCCAGGT*CGGACCG*CGAGGAGGTGGAGATGCCATGCCGACCCACCAGACAAAGCTGGGTATGATAAC	15690–15674
N-for	AAACGGGTTCAGACCTACCAGTATG	86–110
N-rev	TTTGACTGATGTAACACTGGTCTTGAAT	1682–1707
P-for	AGATATCCAGCACAGATGGCAGAAGAGCAGG	1787–1817
P-rev	GCCCTCTAGACTCGAGCTTAAGCATGTGTGATAC	3308–3342
L-for	CAAGCTGGCTAGCGTTTGTCATGGACTCTGTTTC	9010–9043
L-rev	CTGATCAGCGGGTTTTYAGTGRTTTCTAATCAGTGC	15564–15600
vacH-for	TTTCTTCAAAAGT*GTTTAAAC*TGCAATAAACATTGGAA	6985–7022
vacH-rev	TAAGGGA*TTAATTAA*CACAGTTATCATGCCTAAGGCCAATTGA	8922–8964

a Italic letters mean *Not*I, *Bsiw*I, *Pme*I, *Pac*I, and *Cpo*I in sequences. Bold letters refer to a partial sequence of HamRz. Underlined letters refer to a partial sequences of HdvRz.

### Recovery of the recombinant viruses.

Recombinant viruses were recovered as previously described, with modifications (21). Briefly, the BSR-T7 cells were transfected at 80% confluence with 5 μg of pcDNA3.2-HBF or pcDNA3.2-HBF-vacH in combination of 1.0, 0.8, and 0.5μg of pcDNA3.1-N_HBF_, pcDNA3.1-P_HBF_, and pcDNA3.1-L_HBF_, respectively, using X-treme GENE HP transfection (Roche, Basel, Switzerland). The transfected cells were overlaid onto the monolayer canine SLAM-tagged Vero cells at 3 days posttransfection. The cells were maintained at 37°C in DMEM with 2% FBS to observe typical cytopathic syncytia. The recombinant viruses rHBF-1 and rHBF-vacH were propagated and titrated in canine SLAM-tagged Vero cells. Virus titers were performed by limiting dilutions and expressed as the 50% tissue culture infectious dose (TCID_50_) per microliter (mL).

### Characterization of recombinant viruses of rHBF-1 and rHBF-vacH *in vitro*.

The canine SLAM-tagged Vero cells were infected with wtHBF-1, rHBF-1, and rHBF-vacH at an MOI of 0.1 at 80% confluence. The uninfected cells were the control. At 72 hpi, the cells were fixed with 4% paraformaldehyde and permeated with 0.5% Triton X-100, incubated with mouse anti-CDV-NP monoclonal antibody (1:500 dilutions) for 2 h at 37°C, and then stained with fluorescein isothiocyanate (FITC) conjugated goat anti-mouse IgG secondary antibody (1:100 dilutions) for 1 h at 37°C in the dark. After the nucleus was stained with DAPI (4′,6-diamidino-2-phenylindole), fluorescent signals and a bright field were observed under a confocal microscope (Nikon C2, Japan).

The wtHBF-1, rHBF-1, or rHBF-vacH respectively infected canine SLAM-tagged Vero cells infected with wtHBF-1, rHBF-1, or rHBF-vacH were subjected to reverse transcription and PCR (RT-PCR) with specific primers of F3-for/rev (Table 1), to identify the artificial introduction of the *Bsiw*I tag. The *Cpo*I restriction site was uniquely owned by the vaccine H gene, and used to distinguish from the wild-type H gene.

To clarify growth characteristics of each virus, the canine SLAM-tagged Vero cells (1 × 10^6^/well) were infected with the indicated viruses at 0.01 MOI. After 2 h adsorption, the inoculum was removed and the cells were washed twice with medium and further incubated at 35°C. At various times postinfection, the supernatant-associated and cell-associated virus was collected separately and TCID_50_ determined. All these procedures were performed in duplicate.

Eight fields were chosen randomly in canine SLAM-tagged Vero cells infected with wtHBF-1, rHBF-1, and rHBF-vacH at 0.1 MOI at 48 hpi under fluorescent microscope, and the percentages of large syncytia were compared. More than 10 cell nuclei gathered in a syncytium were defined as the large syncytia.

### *In vivo* pathogenicity of the recombinant viruses.

All the ferret experiments were performed as described previously, with modifications (31, 39). Twenty-two CDV serologically negative ferrets were randomly divided into four groups (*n* = 6 in three virus groups; *n* = 4 in mock group) and housed in segregated animal facilities. Three virus-grouped animals were intranasally infected with 10^4.5^ TCID_50_ of wtHBF-1, rHBF-1, or rHBF-vacH, respectively, under general anesthesia. The mock ferrets were intranasally inoculated with 200 μL phosphate-buffered saline (PBS). The bodyweight, rectal temperature, and clinical signs were recorded every day, and the conjunctiva and rectal swabs were collected every 2 days up to 35 days postinfection (dpi). The ill ferrets were euthanized, and the nondiseased animals were euthanized at 35 dpi to collect hearts, lungs, livers, spleens, kidneys, intestines, mesenteric lymph nodes, and brains to detect CDV infection by RT-PCR with universal H primers (forward: 5′-AGACCARGACCTCCCRAGSACGAY-3′ and reverse: 5-GGTATCGGAGGCAATAAATGTCAGC-3′) (40) and quantitate viral RNA loadings by RT-qPCR with specific primers targeted on the CDV N gene (41). The conjunctiva and rectal swabs were also used to detect viral RNA shedding by RT-qPCR assay.

The total immunosuppression of the three indicated viruses was evaluated by the four parameters of lymphopenia, lymphocyte proliferation activity, viremia, and virus neutralization tests (VNTs), as described previously, with minor modifications (6, 31). The EDTA-treated whole blood samples (≥3 ml) were collected from the jugular vein of ferrets under general anesthesia at 0, 3, 7, 10, 14, 21, 28, and 35 dpi, respectively, and 50 μL was used for white blood cell counts with an automated blood analyzer DF55 (DyMind, Shenzhen, China). Peripheral blood mononuclear cells (PBMCs) were isolated from 1 mL EDTA-treated whole blood by Ficoll (TBD, Tianjin, China) gradient centrifugation to determine lymphocyte proliferation activity *in vitro* (34). Briefly, the Ficoll-purified PBMCs (2 × 10^6^/mL) were stimulated or not stimulated with 15 mg/mL phytohaemagglutinin M (PHA). Subsequent bromodeoxyuridine (BrdU) incorporation into proliferating cells was deteced with a cell proliferation BrdU enzyme-linked immunosorbent assay (ELISA) kit (Roche, Basel, Switzerland), and the results were read with a microplate luminometer (Promega-GloMaxNavigator, WI, USA). Cell-associated viremia in PBMCs purified by erythrocyte lysis was quantified by limiting dilutions (23) and expressed as TCID_50_/10^6^ PBMCs. The remaining PBMCs were preserved to assess viral RNA loadings by RT-qPCR.

### Virus neutralization tests (VNTs).

Virus neutralizing antibody (VNA) titers in serum samples collected 0, 3, 7, 10, 14, 21, 28, and 35 dpi were determined in 96-well plates with serial 2-fold dilution mixed with 100 TCID_50_ of rHBF-1 or rHBF-vacH. After incubation at 37°C for 60 min, 50 μL canine SLAM-tagged Vero cells were added. The VNA titers were expressed as the reciprocal of the highest dilution at which no syncytia cytopathic was observed after 5 days.

### Virus infection ability of PBMCs *in vitro*.

The extracted PBMCs (1 × 10^6^) from noninfected ferrets were experimentally infected with wtHBF-1, rHBF-1, or rHBF-vacH for 2 h at an MOI of 0.01, 0.1, or 1, respectively, and then subjected to virus titrations in canine SLAM-tagged Vero cells after PBS washing three times.

To investigate the distinct invasions and potential infection risks of wtHBF-1, rHBF-1, and rHBF-vacH in different host populations, we collected and isolated at least three pieces of PBMCs from 16 kinds of host species mentioned above in “Cells, Viruses, Ferrets, and Blood Samples” to infect with wtHBF-1, rHBF-1, or rHBF-vacH at 0.01 MOI for 2 h, and then titrate in canine SLAM-tagged Vero cells after PBS washing 3 times.

### Statistical analysis.

All data were expressed as the mean ± standard deviation (SD) and analyzed by one-way or two-way ANOVA in SPSS 22.0 (SPSS Inc., Chicago, IL, USA). Statistical significance was considered at *, *p* < 0.05; **, *p* <0.01; ***, *p* <0.001; ****, *p* < 0.0001.

### Ethics statement.

All animal studies and biological sample preparations were approved by the Institutional Animal Care and Use Committee of the Institute of Special Animal and Plant Sciences (ISAPS-2020-062) and complied with the Animal Ethics Procedures and Guidelines.

### Data availability.

The data that support the findings of this study are available from the corresponding author upon reasonable request.
